# Risks to biodiversity from temperature overshoot pathways

**DOI:** 10.1098/rstb.2021.0394

**Published:** 2022-08-15

**Authors:** Andreas L. S. Meyer, Joanne Bentley, Romaric C. Odoulami, Alex L. Pigot, Christopher H. Trisos

**Affiliations:** ^1^ African Climate and Development Initiative, University of Cape Town, Cape Town 7700, South Africa; ^2^ Centre for Biodiversity and Environment Research, Department of Genetics, Evolution and Environment, University College London, London WC1E 6BT, UK; ^3^ Centre for Statistics in Ecology, Environment and Conservation, University of Cape Town, Cape Town 7700, South Africa

**Keywords:** carbon dioxide removal, climate change, climate horizon profiles, Paris agreement, ssp5-3.4-os

## Abstract

Temperature overshoot pathways entail exceeding a specified global warming level (e.g. 1.5°C or 2°C) followed by a decline in warming, achieved through anthropogenically enhanced CO_2_ removal from the atmosphere. However, risks to biodiversity from temperature overshoot pathways are poorly described. Here, we explore biodiversity risks from overshoot by synthesizing existing knowledge and quantifying the dynamics of exposure and de-exposure to potentially dangerous temperatures for more than 30 000 species for a 2°C overshoot scenario. Our results suggest that climate risk to biodiversity from temperature overshoot pathways will arrive suddenly, but decrease only gradually. Peak exposure for biodiversity occurs around the same time as peak global warming, but the rate of de-exposure lags behind the temperature decline. While the global overshoot period lasts around 60 years, the duration of elevated exposure of marine and terrestrial biodiversity is substantially longer (around 100 and 130 years, respectively), with some ecological communities never returning to pre-overshoot exposure levels. Key biodiversity impacts may be irreversible and reliance on widespread CO_2_ removal to reduce warming poses additional risks to biodiversity through altered land use. Avoiding any temperature overshoot must be a priority for reducing biodiversity risks from climate change, followed by limiting the magnitude and duration of any overshoot. More integrated models that include direct and indirect impacts from overshoot are needed to inform policy.

This article is part of the theme issue ‘Ecological complexity and the biosphere: the next 30 years’.

## 1. Introduction

Global average temperatures have already risen by 1.1°C above pre-industrial levels and may rise up to 2.1–3.5°C by 2100 unless stronger mitigation action is taken [1]. Accelerated rates of climate change are triggering numerous ecological changes worldwide, altering species’ phenologies [2], geographical distributions [3], abundances [4] and trophic interactions [5], affecting biodiversity at all levels, from genes to entire biomes. Continued anthropogenic climate change is projected to cause lasting ecological regime shifts and disrupt critical ecosystem services, triggering profound environmental, economic and social losses [6–8]. The extent of these changes will depend on the dynamics of future climate change, including the rate, duration and magnitude of warming [9,10].

The Paris Agreement in 2015 set a long-term goal of limiting global warming to ‘well below’ 2°C above pre-industrial levels and ‘pursuing efforts' to limit warming to 1.5°C. Yet, implementation of greenhouse gas emissions reduction policies by governments remains insufficient to meet these goals [11–13]. This has increased attention on so-called ‘overshoot’ pathways. A temperature overshoot is defined as the temporary exceedance of a specified global warming level followed by a decline to or below that level during a specified time period [14]. Indeed, the IPCC Special Report on global warming of 1.5°C indicates that the majority of emissions scenarios (90%) limiting warming to 1.5°C by 2100 include a temperature overshoot period [15], although more recent scenario approaches produce a wider set of no or very limited overshoot pathways [16]. The magnitude of the overshoot can vary widely, and in most scenarios the duration of the overshoot is at least a decade and typically multiple decades.

While scenarios of future climate change including a temperature overshoot are gaining increasing attention from policymakers, the risks to biodiversity from temperature overshoot pathways are not well described [17]. One key is that biodiversity projections under climate change have typically focused on individual snapshots of risk around 2050 or 2100 for scenarios that either limit global warming to a certain level or have uncontrolled warming reaching 4°C or more by the end of the century. This approach is insufficient to assess risks to biodiversity from overshoot because it lacks the temporal perspective necessary to understand both variation in the magnitude and the duration of the temperature overshoot. In particular, while existing assessments indicate where and when species will be exposed to conditions outside of their thermal niche limits once temperatures exceed global warming targets [18,19], we do not know how long it would take for these species to become ‘de-exposed’ following an overshoot period. This is a critical gap given that the impacts of exposure on individuals, populations and ecosystems are likely to depend not only on how soon and the extent to which their thermal limits are exceeded, but also on the length of time that species remain exposed to these unsuitable conditions. Furthermore, biodiversity climate risk assessments do not typically include risks, such as habitat loss, arising from the negative greenhouse gas emissions approaches required to bring global temperature back down (e.g. large-scale deployment of carbon dioxide removal (CDR) technologies such as bioenergy with carbon capture and storage (BECCS)), although these have been assessed separately [20]. Given current trends in mitigation policies, understanding the potential impact of a temperature overshoot on biodiversity is critical for understanding and managing risks to the diverse ecosystems that humans depend on for their well-being [17].

In this study, we explore risks to biodiversity from temperature overshoot by: (a) describing mean climate trends and extreme events that threaten biodiversity under overshoot pathways; (b) quantifying the temporal dynamics of exposure and de-exposure to temperatures outside the realized niche limits for more than 30 000 terrestrial and marine species under a 2°C temperature overshoot scenario; and (c) synthesizing existing knowledge on direct and indirect overshoot risks to biodiversity, including risk of irreversible change and risk from widespread CDR interventions. We end by outlining important directions for further assessment of overshoot risks and policy considerations for reducing risk to biodiversity.

## 2. The press and pulse of temperature overshoot

Risks to biodiversity from climate change can usefully be conceptualized as an interaction between the impacts of longer term trends in the average climate, such as mean annual temperatures (i.e. climate change ‘press'), and the impacts of shorter-term extreme events such as heatwaves (i.e. climate change ‘pulse’) [21]. Both the press and pulse of climate hazards are projected to increase with the magnitude of global warming. However, the frequency and intensity of climate hazards that threaten biodiversity remain poorly understood for temperature overshoot pathways, particularly for the declining phase of global warming. Moreover, models for biodiversity risk forecasting often do not consider how the duration of the time period over which climate hazards are elevated may impact biodiversity.

Biological systems often exhibit thresholds in their ability to tolerate and recover from acute stresses. However, it is also increasingly appreciated that repeated exposure to climate pulses or prolonged exposure to press conditions that are only moderate in magnitude can overwhelm systems by limiting the time available for recovery or by progressively eroding stress tolerance limits [21]. Isolated, high-magnitude extreme events can then push weakened systems over tipping points between alternative stable states [22]. Thus, the longer populations and ecosystems are exposed to the press of climate trends and the pulse of extreme events under a global temperature overshoot, the more likely they will exceed stress tolerance thresholds, limiting their recovery once the climate returns to pre-overshoot conditions.

The effects of prolonged periods of constant or repeated exposure manifest at all levels of biological organization. At the individual level, recurrent exposures to non-lethal stress may result in decreased performance and, ultimately, mortality [23]. For instance, in maritime pine (*Pinus pinaster*)*,* progressive reductions in growth were observed for consecutive drought events during the 1990 s, 2000 s and 2010 s, the cumulative impacts of which eroded tree resilience and led to recent peaks in mortality [23,24]. The cumulative impacts of multiple stressor events on entire populations have been highlighted by unprecedented forest damage in Central Europe caused by two consecutive years of hot drought [25]. While the drought stress responses in the first year were comparable to previous droughts, the drought stress damage was much greater in the second year, stifling tree growth and leading to unparalleled physiological stress responses. At the ecosystem level, multiple exposures to extreme weather events with insufficient time for recovery between events can tip ecosystems into alternative stable states, as has been observed for coral and forest ecosystems globally [26,27]. For example, projections indicate that coral recovery on the Great Barrier Reef of up to 70% cover within a decade could be achieved, but only if the legacy effects of acute stressors (e.g. cyclones) and the magnitude of chronic stressors (e.g. warming) are reduced [26]. These examples highlight how continued exposure to otherwise bearable stressors can accumulate negative impacts and threaten organisms and ecosystems.

Under overshoot pathways, important differences between regions are expected in the magnitude, duration, and spatial patterning of extreme events. For example, Brazil and western and southern Africa are projected to be particularly at risk of heatwaves under a 1.5°C overshoot scenario [16]. In general, climate-related extreme events are projected to be unevenly distributed across the world under global warming, with tropical and subtropical regions experiencing larger increases in exposure to extreme events compared to higher latitudes [28]. Extrapolating this to an overshoot scenario, we might expect ecological assemblages in these regions to be disproportionately impacted by climate extremes under overshoot. As local climates are expected to warm and cool at different rates from the global mean temperature, it is anticipated that the exposure times of different ecological assemblages will vary regionally.

The climate-related risks from a peak and decline in atmospheric CO_2_ concentrations and associated global warming are also expected to differ between terrestrial and marine realms. For example, surface air temperature responds in a nearly linear fashion to changes in CO_2_ concentration [29]. Therefore, strong reductions in CO_2_ emissions could rapidly stabilize global warming, and an accelerated reduction in atmospheric CO_2_ concentration could reduce air temperatures within a short timeframe. By contrast, global ocean temperatures, dissolved oxygen and pH have a much longer response timescale than temperatures on land. As a result, the ocean is likely to respond more slowly to reductions in atmospheric CO_2_, especially when the overshoot is large [30,31], implying that overshoot pathways may have higher risks for marine ecosystems. Even if global temperatures drop, an increase in slow-onset climate hazards such as ice sheet melt and sea-level rise will continue to last for decades or centuries [32]. Furthermore, the longer response timescale of oceans may have knock-on effects for terrestrial ecosystems. For example, the accumulated heat in the oceans can affect hydrological cycles, thereby reducing rainfall in tropical and subtropical regions and intensifying drying trends in the Amazon, western Africa and Australian regions even decades after a global temperature decrease [33].

**Figure 1.**
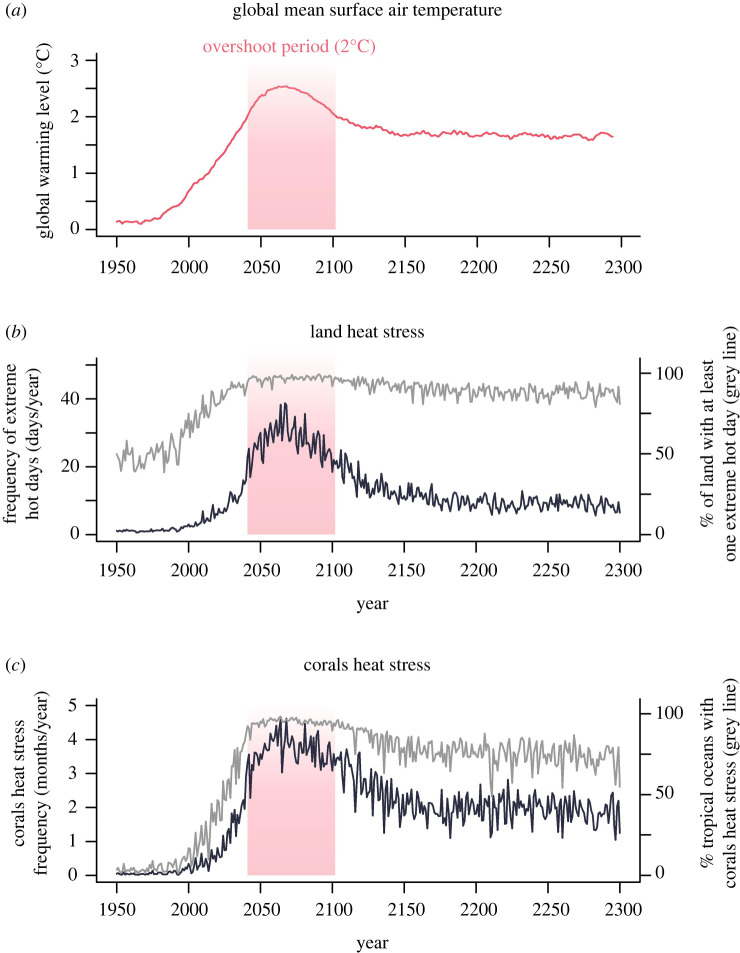
Extreme events will increase in frequency and extent under overshoot, but the rates and magnitude of decrease vary. (*a*) Level of global warming (10-year rolling mean) relative to pre-industrial baseline (1850–1900) calculated based on the median across five global climate models. (*b*) Frequency of extreme hot days per year (black line) and land fraction with extreme hot days (grey line). (*c*) Coral heat stress frequency (black line) and percentage of tropical oceans with heat stress (grey line). An extreme hot day over land was defined as a day with both maximum and minimum temperatures equal to or above their respective 99th percentile for the climatological reference period (1950–1999). The coral heat stress exposure threshold was set at 1°C above the maximum monthly mean sea surface temperature climatology during 1950–1999. The percentage with heat stress represents the percentage of the total land area and percentage of tropical oceans (between 30 °N and 30 °S) affected by stress events. Climate projections are from the SSP5-3.4-OS temperature overshoot scenario (see §3 and electronic supplementary material).

To further explore how temperature overshoot is projected to affect the duration and extent over which potentially dangerous climate conditions occur for biodiversity, we examined the dynamics of heat stress on land and for tropical ocean corals under a 2°C temperature overshoot with strong mitigation and CDR from 2040 (see §3 for scenario description). As global average temperature rises, the frequency and extent of heat stress events increases and then decreases again as the temperature drops (figure 1). However, after peak warming, the rate of decrease in the frequency of extreme events is slower than the projected rate of increase before peak warming and the percentage of both land and tropical oceans exposed to heat stress remains elevated (figure 1*b*,*c*), implying that the risk of these events for biodiversity will last longer than the overshoot period itself. Coral heat stress frequency based on monthly sea surface temperature is projected to decline more slowly after peak warming is reached compared to the frequency of heat stress on land (figure 1*b*,*c*; electronic supplementary material, figure S1). Slower rates of decrease in heat stress frequencies and extent could be explained by the slower rate of cooling after the global temperature peak and by the low magnitude of cooling after the overshoot (i.e. global temperatures only drop slightly below 2°C after 2100).

## 3. Exposure and de-exposure of global biodiversity under an overshoot pathway

To further explore the risks from a temperature overshoot pathway, we projected the temporal dynamics of species exposure to potentially dangerous temperatures under a temperature overshoot scenario (SSP5-3.4-OS). In this scenario, carbon emissions initially follow a high emissions pathway (SSP5-8.5) until 2040, after which a very strong mitigation policy is undertaken, reducing emissions to global net zero by 2070 followed by net-negative emissions until 2140 [34]. After this period, emissions increase gradually, returning to net zero from 2190–2300. Considering a median across the five global climate models used in our analyses (see electronic supplementary material, Methods), this pathway leads to an overshoot of 2°C mean global surface air temperature between 2041 (range across climate models: 2023–2047) and 2102 (2093–2295), with a temperature peak of approximately 2.5°C around 2068 (2059–2081) (figure 1*a*). Beyond 2200 the global warming level decreases to approximately 1.7°C above the pre-industrial period (1850–1900). As temperatures do not return to 1.5°C in this scenario, it was not possible to analyze the risks from a 1.5°C overshoot pathway.

To estimate where and for how long biodiversity would be exposed to potentially dangerous climate conditions we constructed biodiversity climate horizon profiles for species assemblages worldwide (*sensu* [18]). The horizon profile describes when future climate conditions are expected to move beyond the realized thermal niche limit of a species, indicating the cumulative number of species in an assemblage exposed to climate change. Here, we added another feature to the horizon profiles by also estimating de-exposure, which is the number of species projected to again experience suitable climate conditions (i.e. conditions within realized niche limits) after an exposure event.

We built the profiles using data on 30 652 species of amphibians, birds, mammals, reptiles, marine fish, marine invertebrates and seagrasses. We used historical climate data (1850–2014) to estimate the realized thermal niche of each species based on expert range data mapped onto an equal-area 100 km² grid. We defined the realized niche limit as the maximum mean annual temperature experienced by the species across its range between 1850 and 2014. In the following analyses, we treated each grid cell as an assemblage. We classified a species as exposed in the future if it experiences at least five consecutive years of temperatures above its realized niche limits. We classified a species as de-exposed if, after being exposed, the species experiences five consecutive years of temperatures within its niche limits. We acknowledge that the choice of the metric used to classify species as exposed or de-exposed can influence projections of risks to biodiversity. In particular, changing the number of consecutive years that define exposure and de-exposure could influence our estimates. However, previous analyses of global warming scenarios have returned similar results when defining exposure using 5- or 20-year windows [18]. Ideally, metrics related to timing of exposure could be tailored to the life history of each species, but this is not yet feasible when assessing exposure for thousands of species globally.

**Figure 2.**
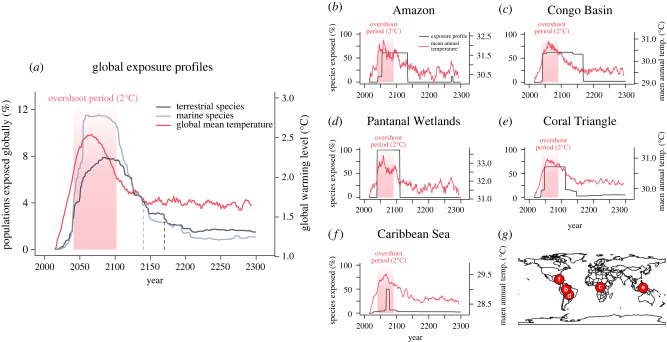
The exposure and de-exposure of biodiversity under a 2°C temperature overshoot pathway. (*a*) Profile showing the percentage of species populations globally exposed to temperature beyond species' realized niche limits (a population is a species occurrence within a grid cell). The black and grey lines show the percentage of populations exposed to unprecedented temperatures over time for terrestrial and marine species, respectively. The red line shows the level of global warming above the pre-industrial baseline (1850–1900). The red shading indicates the duration of the 2°C global temperature overshoot period. The vertical dashed lines indicate when the percentage of exposed species populations returns to pre-overshoot levels for terrestrial and marine species. In (*a*) the median value across five climate models is shown. (*b*–*f*) Examples of exposure profiles for iconic ecosystems: (*b*) Amazon, (*c*) Congo Basin, (*d*) Pantanal Wetlands, (*e*) Coral Triangle and (*f*) Caribbean Sea. Black lines show the profile of exposure and de-exposure at a site. Red lines show the local temperature trends. Exposure profiles and temperature trends show data from a single cell within each ecosystem. Data for (*b–f*) are from a single run of the NASA Goddard Institute for Space Studies Model (GISS-E2-1-G). (*g*) Locations of the five iconic ecosystems in (*b–f)*.

Although the global mean air temperature trend shows a clear peak for the overshoot scenario (figure 2*a*, electronic supplementary material, figure S2), local temperature trends can show high variability. Consequently, a species at a given site can potentially be exposed, and subsequently de-exposed, multiple times. Therefore, when constructing the horizon profiles for a site we counted all events of exposure and de-exposure for each species at that site. From the profiles, we derived seven exposure and de-exposure metrics, including (i) maximum exposure; the maximum percentage of species in a state of exposure at any time between 2015 and 2300, (ii) final exposure; the percentage of species in a state of exposure at 2300, (iii) total de-exposure; the decrease between maximum and final exposure as a percentage of the maximum exposure (i.e. a larger value indicates a higher % of species de-exposed), (iv) timing of exposure; the median year in which exposure events occur, (v) abruptness of exposure; the percentage of all species exposure events between 2015 and 2300 that occur within the decade of maximum exposure, and (vi) abruptness of de-exposure; the percentage of all species de-exposure events that occur within the decade of maximum de-exposure. Finally, we calculated (vii) the duration of overshoot for biodiversity exposure. We did this by quantifying the percentage of species in an assemblage exposed at the beginning of the global 2°C overshoot period and then calculating how long it would take for the exposure levels to return to (or below) that level of exposure. To assess whether the duration of overshoot for biodiversity would last longer than the global 2°C overshoot period we subtracted the duration of the global 2°C overshoot from the duration of overshoot for biodiversity exposure. A detailed description of the Methods is provided in the electronic supplementary material, Methods.

**Figure 3.**
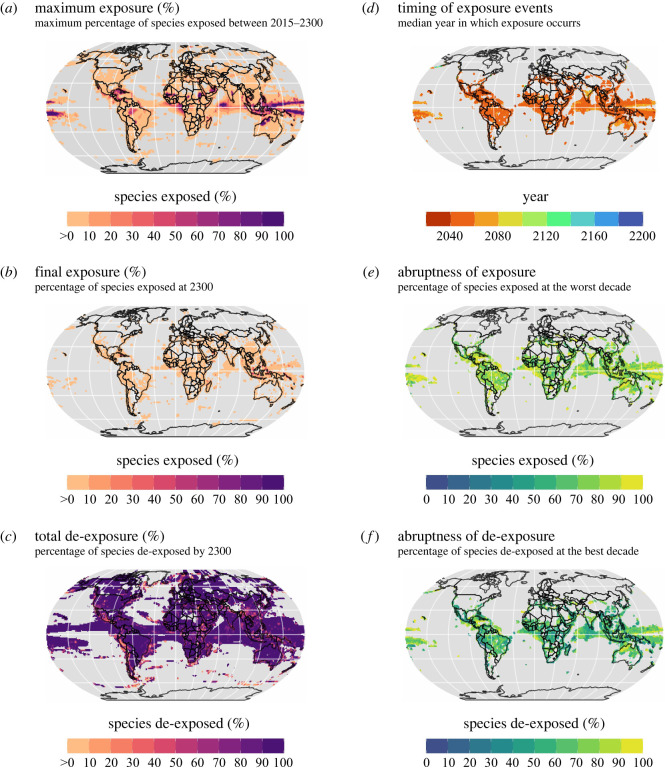
Global variation in the magnitude, timing and abruptness of exposure and de-exposure of biodiversity for a 2°C temperature overshoot scenario. (*a*) Maximum percentage of species in a grid cell exposed to temperatures outside of their historical thermal niche limits. (*b*) Final exposure at 2300. (*c*) Total de-exposure (decrease between maximum and final exposure as a percentage of the maximum exposure). (*d*) Timing of exposure. (*e*) Abruptness of exposure. (*f*) Abruptness of de-exposure. Maps show data from assemblages with five or more species exposed. The maps show the median value across five global climate models.

We found that the projected magnitude of exposure under the 2°C temperature overshoot scenario is greater for marine compared to terrestrial biodiversity. Globally, during the global 2°C overshoot period, the exposure of biodiversity to temperatures beyond species' historical limits peaks at an average maximum of 11.5% for marine species populations (range across climate models: 6.4%–19.3%) and 8% for terrestrial species populations (5.2%–13.3%) (figure 2*a*; electronic supplementary material, figure S2). For both marine and terrestrial realms, the populations exposed to potentially unsuitable temperatures are mostly concentrated in the tropics (figure 3*a*). Regions most at risk are the Indo-Pacific, Central Indian Ocean, Northern Sub-Saharan Africa and Northern Australia, where greater than 90% of species in many assemblages are projected to be exposed in the future (figure 3*a*). In species-rich regions such as the Amazon and Caribbean, the magnitude of exposure reaches greater than 50% in many assemblages (figure 3*a*).

At the global scale, for terrestrial biodiversity, exposure peaks on average 15 years after peak global warming, whereas for marine biodiversity, exposure peaks on average 8 years after peak global warming (figure 2*a*). The timing of exposure also differs regionally. Across 80% of sites, the median time of exposure occurs at least 10 years before the time of peak global warming, for 18% of sites it is within the decade of peak global warming, and for 2% of sites it occurs at least 10 years after peak global warming (figures 2*a* and 3*d*). Interestingly, substantial lags in the timing of exposure are projected in a few regions, due to local warming lagging behind global warming. For example, parts of India and the Aleutian Islands (northernmost Pacific) have median exposure times 30 and 58 years after the time of peak global warming, respectively (figure 3*d*). This indicates that even as global temperatures decline to reach pre-overshoot levels, increases in exposure will still continue to occur for some local assemblages, as local climates warm and cool at different rates from the global mean temperature.

Globally, for both terrestrial and marine biodiversity, exposure increases rapidly after the beginning of the overshoot, with the increase being particularly rapid in the oceans (figure 2*a*). By contrast, for both marine and terrestrial biodiversity the rate of de-exposure after peak global warming is much more gradual (figure 2*a*). After first exceeding 2°C in the 2040s, the global warming level returns to 2°C around 2100 (a temperature overshoot of 60 years) but it takes a further 40 years for global marine and 70 years for global terrestrial biodiversity exposure levels to decline to the same exposure levels as the 2040s (figure 2*a*)—making the duration of exposure for global marine and terrestrial biodiversity 66% and 115% longer, respectively, than the temperature overshoot.

**Figure 4.**
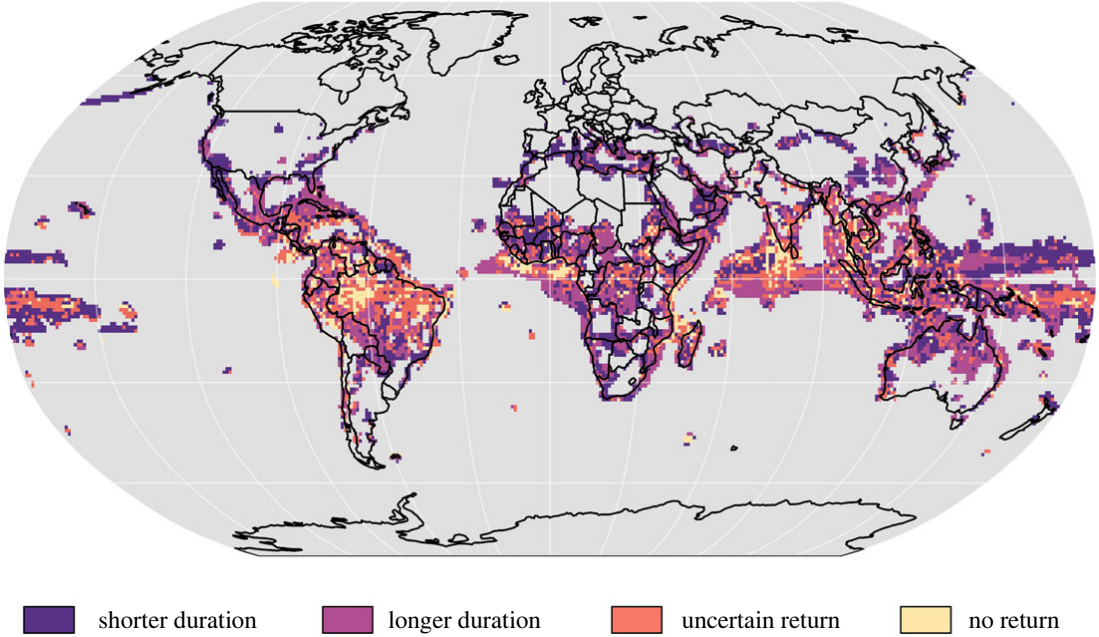
Regions of uncertain or no return to pre-overshoot levels of exposure for local biodiversity. Yellow shows assemblages for which most climate models projected no return to pre-overshoot exposure levels for biodiversity (8% of sites). Orange indicates assemblages in which projections were uncertain regarding a potential return—that is, half of the climate model projections indicated the assemblage would not return to pre-overshoot exposure levels (19% of sites). Light purple indicates assemblages where the duration of overshoot in the exposure of local biodiversity is projected to be longer than the duration of the 2°C global temperature overshoot and dark purple indicates where the duration of overshoot in the exposure of local biodiversity will be shorter. The map shows data from assemblages with five or more species exposed. The reported results are the median values across four climate models (see electronic supplementary material, Methods).

In accordance with the global trends, at the scale of individual sites, exposure of biodiversity typically increases more abruptly than it decreases (figure 3*e*,*f*). The median abruptness of exposure for species assemblages is 75% (range across climate models: 63%–82%) for terrestrial and 84% (73%–91%) for marine assemblages, while the median abruptness of de-exposure is lower at 54% (47%–60%) for terrestrial and 71% (50%–85%) for marine assemblages. For 35% of sites, the period of overshoot for biodiversity exposure is projected to be longer than the global temperature overshoot (figure 4). Of even more concern is that for 19% of sites, there is disagreement among climate models regarding whether or not the percentage of exposed species would return to pre-overshoot levels, and across 8% of sites, all models project that exposure will not return to pre-overshoot levels. Regions of uncertain and no return to pre-overshoot levels of exposure mainly include the tropics, especially the Amazon, the tropical African coast, Indian Ocean, Southeast Asia and the Indo-Pacific (figure 4). By contrast, the duration of overshoot in exposure for local biodiversity is shorter than the global temperature overshoot across 38% of sites, mostly in extra-tropical regions (figure 4). Lastly, although total de-exposure is predicted to be high in most regions (figure 3*c*), most assemblages will continue to have some species exposed by 2300 (figure 3*b*). Taken together, these results suggest that the potential negative stresses for biodiversity from a temperature overshoot will arrive suddenly, but decrease more gradually. These results highlight that unless temperatures are rapidly reduced to well below 2°C after the overshoot, conditions unsuitable for many species will persist, implying long-term disruption to ecosystems. They also highlight the variation in the duration of elevated exposure across sites as another important dimension of risk from a temperature overshoot, in addition to the magnitude of exposure.

A few non-exclusive hypotheses might explain why the time to de-exposure is disproportionately longer both globally (figure 2*a*) and locally (figure 4). First, delayed de-exposure might be associated with trends in mean global surface air temperature. For example, global warming rates are slightly faster than cooling rates, which could explain why exposure decreases at a slower rate. However, there is a key difference between global and local de-exposure trends. While at a global scale species exposure returns to pre-overshoot levels (figure 2*a*), at local scales 8% of the sites are projected not to return to pre-overshoot levels, with a further 19% of the sites exhibiting disagreement among climate models. Such differences might be explained by particular site-scale climatic trends, which may also play an important role in explaining the delayed de-exposure of biodiversity following the overshoot. Second, yearly variation in temperatures may also influence local, and consequently global de-exposure. Our criterion to classify a species as exposed is five consecutive years under unsuitable climates. To become de-exposed, the same species must experience five consecutive years under suitable climates. During the warming phase of the overshoot, temperature increases at faster rates and the yearly variation is relatively less pronounced. Hence, it is more likely that a species will experience five consecutive years of unprecedented temperature at this phase. During the cooling phase of the overshoot, the yearly temperature variation is higher, which means that even though the global temperature is decreasing, there is a higher probability that a species will experience at least one year with inimical temperatures within the five-year window used to detect de-exposure—highlighting how the interval between exposure events can be crucial to species recovery, as discussed above.

Our analysis represents a first step in understanding risks to biodiversity under a temperature overshoot scenario. Here, our focus is on risks of exposure to *in situ* populations and assemblages, and we do not attempt to model the dynamics of extinction or how species may colonize new sites. We also acknowledge that our estimates do not account for local microclimate heterogeneity, evolutionary adaptation or that species may have wider fundamental than realized niche limits, all of which could reduce the adverse effects of exposure. Many of these limitations are common to large-scale biodiversity models, but despite this, there is no evidence that these models systematically overestimate risks to biodiversity [35]. Indeed, other factors that we do not consider, such as local adaptation or cascading disruption to ecological interactions, are likely to increase risks from exposure [36]. Understanding the sensitivity of projected risks to biodiversity from overshoot to these assumptions is a priority for future research.

It is also important to recognize that some of our results may be scenario-dependent. Our projection that some assemblages will never return to pre-overshoot exposure levels is probably influenced by the gradual increase of emissions in the SSP5-3.4-OS scenario going from net-negative emissions during 2070–2140 back to net zero emissions by 2190. If emissions instead remained net-negative after 2190, it is probable that more assemblages would return to pre-overshoot levels before 2300. However, the feasibility of very strong carbon dioxide removal (tens to hundreds of GtCO_2_) deployed rapidly and maintained over long time periods remains highly uncertain [13,37,38]. Thus, our results indicating more rapid exposure than de-exposure are likely applicable to a broad range of temperature overshoot scenarios, especially those with more realistic CDR deployments. Moreover, a prolonged period with high rates of large-scale CDR deployment would introduce substantial additional risks for biodiversity (some of which are reviewed below).

## 4. Fast- and slow-onset tipping points, irreversible ecosystem transformation and extinction

Our analysis assumes that following the exposure of species to conditions beyond their niche limits, they can be de-exposed if temperatures subsequently decline, implying that climate impacts on biodiversity are reversible. However, the effects of exposure on species that become extinct will be irreversible. It is projected that even at a warming level of 2°C, approximately 10% of species globally are at risk of extinction [39]. For communities that become extinct at the regional scale, recolonization of habitat following climate overshoot may be limited or very slow, as observed for forests in Europe since the last ice age [40]. Catastrophic ecological changes can also occur abruptly once a critical tipping point in the external environment is exceeded, potentially causing irreversible transformations of systems into alternative stable states. Under these circumstances, de-exposure is irrelevant, as species would not recover even if the conditions became suitable again in the future. These changes may result in a cascade of processes that create positive climate feedback loops that reduce the likelihood of a return to a given global warming level, such as through the release of thousands of Gt of carbon from high-carbon ecosystems as a result of accelerated forest fires, drying of peatlands and thawing of permafrost [41].

When a tipping point occurs relatively quickly after exceeding some threshold—on the timescale of years to decades—it represents a fast-onset tipping point [42]. Few empirical studies have explicitly projected tipping points under temperature overshoot. One study explored whether changes in climate lead to critical transitions in an alpine forest system as well as whether this transition could be reversed by reversing the climate forcing (i.e. a hypothetical overshoot scenario). Projections indicated that a tipping point whereby a conifer-dominated landscape was transformed into a landscape of smaller broadleaved species occurred at a warming level of 2°C. However, when the simulated warming exceeded 2°C and the climate forcing was reversed, the capacity for the system to recover was limited, with impacts on forest structure and species composition that were irreversible even after 1000 years [43]. In our analysis of biodiversity exposure and de-exposure, a substantial portion of terrestrial communities in the Amazon Forest and southeast Asia are projected not to return to within realized niche limits even by 2300, implying that transformation into an alternative stable state is a very possible outcome of temperature overshoot for these ecosystems. This also corroborates predictions of the rapid collapse of the Amazon under short high-magnitude overshoot once thermal thresholds are exceeded on shorter timescales [42].

Due to the inherent inertia of the response of some systems, the transformation of systems into alternative stable states could also occur at a significantly slower rate—at the centennial scale—representing a slow-onset tipping point. Examples include the melting of ice sheets [43,44] and thawing of Arctic permafrost [45]. For many tipping points, particularly slow-onset tipping points, it is possible for thresholds to be exceeded without the system immediately tipping into an alternative stable state [42,45].

Both the duration and magnitude of the overshoot are important contributors to the collapse of fast- and slow-onset systems. For example, modelling the collapse of the Atlantic meridional overturning circulation (AMOC)—a slow-onset system—under overshoot indicated that shorter high-magnitude overshoot allowed for sufficient recovery of the system, whereas a longer duration of low-magnitude overshoot severely weakened the ocean flow rate, resulting in the sustained collapse of the system [42]. The strengthening of the AMOC during the deglacial transition of the Bølling–Allerød warm period (14.7–12.9 kya) was associated with abrupt changes in terrestrial climate, water availability and vegetation composition [46]. Simulations of Arctic permafrost decomposition rates even under low climate overshoot scenarios (climate forcing peak in 2050 and reversal by 2065) indicate irreversible transformations into alternative states for high northern latitude permafrost and the terrestrial carbon cycle [45]. The long-term impacts of overshoot on the region are proportional to the magnitude of the overshoot, with potential multiple steady states possible for the region in the future. For the high northern latitudes, small-scale overshoot may already be altering the steady state, implying that the emissions targets over the next three decades could be pivotal in dictating the future Arctic environment.

Ocean ecosystems are projected to respond slowly to reduced atmospheric CO_2_ under CDR following overshoot, especially when the magnitude of the overshoot is high or the duration is long [30]. Our projections of heat stress frequency in corals under overshoot emphasize that heat stress in the ocean could be more persistent than on land (figure 1*b*,*c*; electronic supplementary material, figure S1). Our exposure analysis also found that a greater percentage of marine populations than terrestrial populations would be exposed to unprecedented temperatures under an overshoot. Abruptness of exposure is also higher for marine species, although de-exposure is also higher. Consequently, the return to pre-overshoot exposure levels occurs earlier in the marine compared to the terrestrial realm. Climate overshoot is projected to result in greater ocean acidification than a stabilization pathway, with ocean pH remaining low for up to 20 years after the overshoot simulation stabilizes to atmospheric CO_2_ trends [31]. This is concerning for corals, which face major degradation in the next 20–30 years even with moderate warming under an intermediate emissions scenario (RCP4.5) [47]. In our analysis, a significant proportion of marine communities are predicted never to return to pre-overshoot exposure levels once global climate stabilizes, particularly in West Africa and the South Asian Indian Ocean (figure 4). These findings suggest that the consequences of overshoot for the marine environment are likely to be long-lasting, with detrimental and potentially irreversible impacts on biodiversity.

The rapid increase in exposure under overshoot detected in our analysis for both marine and terrestrial communities suggests that exposure will be sudden (figures 2*a*, and 3*e*). Rapid onset of overshoot could cause local extirpation in climate-sensitive organisms, resulting in population bottlenecks and reduced diversity [48,49], which could hinder adaptation and persistence. A high rate of onset of warming, rather than the peak temperature, resulted in the mass mortality of coral reef fish [48], implying that rapid increases in temperature under overshoot could trigger mortality events. Mass die-offs are expected with sudden exposure to unprecedented temperatures, as reported for flying-foxes in Australia [50], birds and fruit bats in South Africa [51], North Atlantic seagrass [52,53], white sea sponges [54] and Mediterranean corals [55]. Limiting both the magnitude and duration of exposure of systems to increased climate extremes under global temperature overshoot is thus critical for reducing the progressive and sudden erosion of stress tolerance limits and therefore severe and irreversible biodiversity loss.

## 5. Risks from land-use change for temperature overshoot

Scenarios that overshoot the long-term temperature target of the Paris Agreement and then bring global warming levels back below 2°C rely on large-scale deployment of CDR, such as BECCS and afforestation, which themselves present substantial risks to biodiversity [56]. The vast land requirements of afforestation and BECCS for climate mitigation under overshoot could severely negatively impact biodiversity and ecosystem functioning. For instance, bioenergy plantations and forests could compete for the same land area and reduce the land available for biodiversity conservation and food production. The land-use changes associated with afforestation would have negative consequences for native biodiversity if trees replace naturally unforested ecosystems, such as grasslands and savannahs (these ecosystems are often incorrectly identified as degraded and thus as available land for tree planting) [57]. Further negative consequences for biodiversity could result from afforestation reducing runoff into streams, depleting groundwater and facilitating the spread of invasive species [57,58]. The cultivation of bioenergy crops is also associated with greater water consumption and nitrogen application [20]. Furthermore, converting naturally unforested land into forest may increase warming, such as by altering albedo (forests absorb more incoming solar radiation than grasslands) or by increasing the release of greenhouse gases should forests burn [57–59]. A climate model for which temperature overshoots the 1.5°C target until mid-century and then declines rapidly to emulate net negative emissions to meet 1.5°C by 2100 (theoretically achieved through CDR technology) is predicted to have significant negative consequences for the hydrological cycle, ocean circulation, regional surface warming, sea ice and sea levels [31].

Overshoot scenarios are also expected to have larger negative impacts relating to crop growth duration, drought and impacts from climate extremes than scenarios with no overshoot [16]. Reduced agricultural productivity under overshoot could further transform natural tracts of land for agriculture. This transformed land is unlikely to be returned to its original state once temperatures stabilize again. Overshoot-related habitat fragmentation or transformation could reduce population genetic diversity or cause the loss of beneficial adaptive alleles, thereby hindering adaptive capacity. Overshoot is also projected to cause an 18% higher global mean steric sea-level rise [31]. Saltwater intrusion into coastal areas that were previously not saline could result in the inland retreat of non-adapted plants, difficulties in seed and seedling establishment, mass die-backs of coastal forest not tolerant of salt and the range expansion of invasive species tolerant of salinity at the expense of indigenous species, all of which could lead to irreversible ecosystem transformation. The clearing of land as human settlements are displaced and move further inland could further exacerbate this. Increased sea level rise will also have long-term and irreversible consequences, as some of the heat would enter the deep sea and only equilibrate centuries later [31].

## 6. Summary and future prospects

Avoiding temperature overshoot is a priority for reducing climate change risks to biodiversity, followed by limiting the magnitude and duration of any overshoot. Biodiversity risks are likely to be exacerbated the longer species are exposed to the increasing press of climate trends and pulse of extremes under increased global warming. Without rapid and deep emission cuts, global warming will exceed the temperature targets of the Paris Agreement around the middle of this century. Temperature overshoot scenarios that return warming to below 1.5 or 2°C by 2100 are thus becoming increasingly policy-relevant. However, as demonstrated by our results, increased climate-related risks to biodiversity from exceeding 2°C may remain for decades to centuries longer than the period of temperature overshoot. For many ecosystems, thresholds for irreversible impacts are expected to be exceeded even under a short overshoot duration. To reduce the risks to biodiversity, it is imperative that rapid and deep emissions cuts are implemented immediately. The early timing of this mitigation action is critical for reducing long-lasting negative impacts on biodiversity because the magnitude and duration of any temperature overshoot may be grossly underestimated [60,61], due to high uncertainty about the feasibility of any large-scale deployment of CDR approaches. Moreover, large-scale CDR deployment to rapidly reduce a substantial temperature overshoot would likely involve strong trade-offs with biodiversity protection, water security and agricultural production. Given the hard policy decisions ahead, more integrated models that include both direct and indirect risks from temperature overshoot are required in order to make more robust projections of overshoot risk to biodiversity.

## Data Availability

The data and codes used in the analyses are available from FigShare: https://doi.org/10.6084/m9.figshare.19874176.v1 [62]. Electronic supplementary material is available online [63].
